# Safety and effectiveness of lanthanum carbonate for hyperphosphatemia in chronic kidney disease (CKD) patients: a meta-analysis

**DOI:** 10.1080/0886022X.2021.1986068

**Published:** 2021-10-04

**Authors:** Lijuan Zhao, An Liu, Guoshuang Xu

**Affiliations:** a Department of Nephrology, Xijing Hospital, The Fourth Military Medical University of People’s Liberation Army, Xi’an, China; b Outpatient Department, Xi’an Children’s Hospital, Xi’an, China

**Keywords:** Chronic kidney disease, hyperphosphatemia, lanthanum carbonate, efficacy, safety

## Abstract

**Objective:**

The aim of this study was to determine the efficacy and safety of lanthanum carbonate (LC) versus calcium salts, non-LC phosphate binders (PBs), sevelamer, or placebo in patients with chronic kidney disease (CKD).

**Materials and methods:**

A literature search on PubMed, Embase, and Cochrane Library databases was conducted up to 18 June 2021. Data acquisition and quality assessment were performed by two reviewers. Meta-analysis was performed to evaluate the serum biochemical parameters, adverse events, and patient-level outcomes of LC, non-LC PBs, and sevelamer for hyperphosphatemia in patients with CKD. Heterogeneity across studies was assessed utilizing the *I*^2^ statistic and *Q*-test, and a random effect model was selected to calculate the pooled effect size.

**Results:**

A total of 26 randomized, controlled trials and 3 observational studies were included. Compared to the other groups, better control effect of serum phosphorus (RR = 2.68, *p* < 0.001), reduction in serum phosphorus (95%CI = −1.93, −0.99; *p* < 0.001), Ca × P (95%CI = −13.89, −2.99; *p* = 0.002), serum intact parathyroid hormone levels (95%CI = −181.17, −3.96, *p* = 0.041) were found in LC group. Besides, reduced risk of various adverse effects, such as hypotension, abdominal pain, diarrhea, dyspepsia, and a score of coronary artery calcification were identified with LC in comparison to calcium salt, non-LC PBs, or placebo group. Significantly lower risk in mortality with LC treatment vs. non-LC PBs was observed, while no significant difference was identified between LC and calcium salt groups.

**Conclusion:**

LC might be an alternative treatment for hyperphosphatemia in patients with CKD considering its comprehensive curative effect.

## 1. Introduction

Chronic kidney disease (CKD) is a significant health problem worldwide [1]. Hyperphosphatemia is very common and harmful in patients undergoing maintenance dialysis [2,3]. Previous studies suggested that hyperphosphatemia is an independent risk factor for surrogate clinical endpoints like morbidity and mortality for patients with CKD [4], or the development of coronary artery calcification in peritoneal dialysis patients [5]. Evolving effective agents is an essential part of CKD therapy.

Currently, the management of hyperphosphatemia in patients with CKD is depending on intestinal phosphate binders (PBs), including non-calcium-based binders or calcium-based agents [6]. Although effective in reducing serum phosphorus levels, security issues need to be considered and explored when choosing which one to use. Gastrointestinal adverse events and cardiovascular disease are major problems for the complication of these PBs. The progression of vascular calcification of media is considered to be the main influencing factor [7]. A prospective randomized study in hemodialysis patients reveals that excessive use of calcium carbonate is likely to cause hypercalcemia, which is continuously related to progressive arterial calcification and decreased trabecular bone density within 2 years of observation [8]. In addition, a meta-analysis has found that sevelamer, a nonabsorbed, calcium- and metal-free dietary PB, does not produce sustained superior biochemical outcomes in comparison to calcium-based therapies [9]. However, another meta-analysis of 11 randomized trials (4622 patients) suggests that non-calcium-based PBs are superior to calcium-based PBs in decrease the all-cause mortality risk in patients with CKD [10]. Yet to date, the impact of these agents on patient-level outcomes is still a controversial issue.

Lanthanum carbonate (LC), as a new non-calcium-based PB, is used to treat hyperphosphatemia in patients with CKD through binding phosphate via its trivalent cation [6,11]. Reportedly, healthy individuals receiving a dose of 3000 mg/day of LC can reduce urinary phosphorus excretion [12]. In addition, a multicenter, randomized, and double-blind study showed that LC is a well-tolerated and efficacious oral PB with mild adverse effects for hemodialysis and patients with CKD [13]. To further analyze the efficacy and safety of LC, the present meta-analysis was performed via comparing the effects of LC on serum biochemical parameters, various adverse events, and patient-level outcomes versus calcium salts (calcium acetate and calcium carbonate), sevelamer hydrochloride (SH), non-LC PBs (PBs) or placebo.

## 2. Materials and methods

### 2.1. Data sources

A systematic search strategy was performed and LC relevant clinical articles were obtained from PubMed (http://www.ncbi.nlm.nih.gov/PubMed), Embase (http://www.embase.com), and Cochrane Library (http://www.cochranelibrary.com/) electronic databases. Following terms including (Lanthanum carbonate) OR fosrenol OR (dilanthanumtricarbonate) OR (lanthanum carbonate hydrate) AND (CKD OR (chronic renal failure) OR (chronic kidney disease) OR (chronic nephropathy) OR hemodialysis OR (Peritoneal dialysis) OR (end-stage renal disease) OR ESRD) were used for searching. Based on titles and abstracts, the relevant studies were screened by two investigators separately (LJZ and AL), and reviewed by a third one (GSX). Any disagreement between the two researchers was resolved by a third person review (Kappa = 0.895, Se = 0.024, *p* < 0.001). The literature was published in English and the deadline for the literature search was 18 June 2021. The details of the retrieval strategy are shown in Supplementary Tables 1–3.

### 2.2. Inclusion and exclusion criteria

The inclusion criteria of the study were as follows: (a) the subjects of this study were CKD patients with hyperphosphatemia, including those who underwent hemodialysis, peritoneal dialysis, or did not undergo dialysis; (b) patients in the treatment group were treated with LC; (c) patients in the control group were treated with calcium-phosphorus binders (e.g., calcium acetate or calcium carbonate, etc.), non-calcium-phosphorus binders (e.g., sevelamer, iron, etc.), placebo or without using phosphorus reducing agents; (d) randomized controlled trial (RCT) or observational research; and (e) providing information regarding the effectiveness and safety outcomes, such as blood phosphorus control, blood phosphorus levels, alkaline phosphatase, death, as well as adverse reactions. The exclusion criteria of the study were as follows: (a) studies that could not be used for statistical analysis due to incomplete data; (b) non-original articles: including review, letter, and comments; and (c) duplicated publications.

### 2.3. Data extraction and quality assessment

Data extraction was conducted independently by two authors (LJZ and AL) and reviewed by a third one (GSX). The disagreements between two researchers were resolved by a third person review (Kappa = 0.638, Se = 0.041, *p* < 0.001). The following data were extracted and recorded: the first author of the literature, the year of publication, study type, age and gender of the subjects, sample size, therapy intervention, follow-up period, and the outcome data of patients. Differences were resolved by discussion.

Cochrane risk of bias assessment tool was used to evaluate the quality of the included RCT study [14]. Evaluation items included sequence generation, allocation concealment, blinding of participants and personnel, blinding of outcome assessment, incomplete outcome data, selective outcome reporting, and other biases. Meanwhile, the methodological quality of the observational study was assessed by the quality evaluation criteria provided by the Newcastle–Ottawa Scale (NOS), including the evaluation of exposure selection, comparability, and outcome, with a full score of 9 points [15]. Importantly, disagreements regarding the quality evaluation were solved after a group discussion with the third author.

### 2.4. Statistical analysis

All statistical analysis was performed by Stata 11.0 software. Relative ratio (RR) and 95% confidence interval (CI) were used for the categorical variables. However, weighted mean difference (WMD) and 95% CI were used as the combined index for the continuous variables. Random effect models were used to merge the outcomes of all studies. In addition, Cochran's Q statistics and *I*^2^ test were used for the heterogeneity test [16]. Heterogeneity was significant among studies if *p* < 0.05 or/and *I*^2^>50%. While *p* ≥0.05 and *I*^2^≤50% indicated there was no significant heterogeneity. Furthermore, subgroup analysis was used to explore the effects of age (<60 years or ≥60 years), region (Asian or western), and sample size (<100 or ≥100) on the merger results. Publication bias was tested by Egger's test, and *p* <0.05 was considered significant.

## 3. Results

### 3.1. General characteristics of selected literature

**Figure 1.**
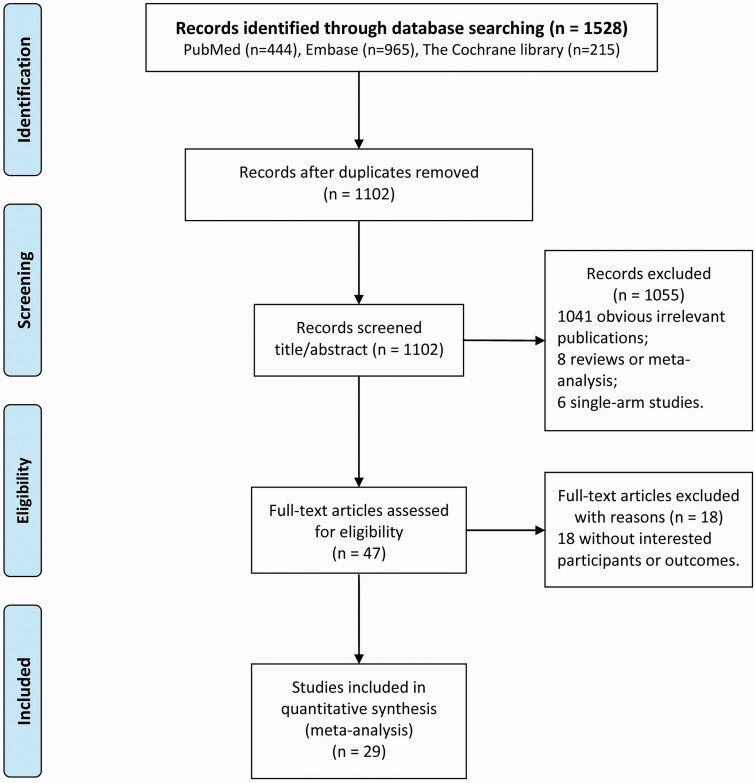
Flow diagram of the study selection process.

The flowchart of literature search and study selection is displayed in Figure 1. A total of 1528 potential articles were relevant to the search terms (444 from PubMed, 965 from Embase, and 215 from Cochrane library). After eliminating 426 duplicate literature, 1041 obvious irrelevant publications, 8 reviews or meta-analysis, 6 single-arm studies, 18 articles without interested participants or outcomes, a total of 29 eligible studies [13,17–44]. were included for this meta-analysis.

The characteristics of included studies are listed in Table 1. In total, 29 studies including 26 RCT and 3 retrospective cohort studies were included. The publication year of included studies ranged from 2003 to 2021, and studies were conducted in various countries, including China, Japan, Korea, USA, UK, India, Macedonia, and Australia. The duration of follow-up ranged from 4 weeks to 5 years. Notably, there were four control groups according to the type of binders used, including calcium salts, sevelamer, non-LC PBs, and placebo. For each study, no significant differences in baseline information between control and experimental groups were found.

**Table 1.** Characteristics of the studies included in the meta-analysis.

Author	Year	Area	Design	Patients	Define of hyperphosphatemia	Follow-up duration	Intervention, mg/d	*N*, M/F	Age (years)	Duration of dialysis (years)
Hutchison, AJ	2005	European	RCT	HD	Serum phosphate, >5.58 mg/dL	6 ms	LC (375–3000) CC (1500–9000)	510, 341/169 257, 164/93	57.0 ± 14.3 58.4 ± 13.3	42.9 ± 39.0 ms 43.8 ± 43.9 ms
Spasovski, GB	2006	Macedonia	RCT	ESRD, Newlydialysis	Serum phosphate, >5.6 mg/dL	1 years	LC (750–3000) CC (1000–4000)	12, 7/5 12, 7/5	55 ± 10 57 ± 10	<12 weeks <12 weeks
Shigematsu, T (a)	2008	Japan	RCT	CKD, HD	Serum phosphate, >5.6 mg/dL	8 weeks	LC (750–2250) CC (1500–4500)	126, 87/39 132, 87/45	58.8 ± 10.5 56.1 ± 11.5	9.8 ± 7.3 9.7 ± 7.2
Scaria, PT	2009	India	RCT	CKD 4, HD	Serum phosphate, >5.6 mg/dL	4 weeks	LC (1500)	20, 13/7	49.9	NR
							CA (2000)			
										
Toussaint, ND	2011	Australia	RCT	ESRD, HD	Serum phosphate, >5.0 mg/dL	18 ms	LC (3088 ± 744) CC (7269 ± 2017)	22, 12/10 23, 17/6	56.0 ± 15.2 58.8 ± 14.9	38.8 ± 42.9 42.3 ± 46.6
Toida, T	2012	Japan	RCT	HD	NR	3 ms	LC–first (750) CC–first (1500)	25, 15/10 25, 15/10	65.2 ± 13.8 65.9 ± 8.9	7.1 ± 6.8 6.5 ± 5.2
Lee, YK	2013	Korea	RCT	ESRD, CAPD	Serum phosphate, >5.6 mg/dL)	24 weeks	LC (1500) CC (3000)	20, 11/9 30, 11/19	48.3 ± 11.1 51.8 ± 11.6	55.73 ± 48.09 ms 69.67 ± 53.89 ms
Chang, YM	2017	Taiwan	RCT	CKD, HD	Serum phosphate, >6.0 mg/dL	24 weeks	LC (1644 ± 584) CC (3375 ± 1299)	13, NR 12, NR	56.62 ± 11.51 61.17 ± 7.76	74.46 ± 61.79 ms 73.75 ± 43.76 ms
Zhang, C	2017	China	RCT	DM, HD	Serum phosphate, >5.6 mg/dL	1 years	LC (750–2250) CC (1500–3750)	46, 33/13 46, 36/10	48.2 ± 8.9 51.0 ± 9.5	3.73 ± 1.46 5.06 ± 1.31
Fujii, H	2018	Japan	RCT	ESRD, Newly dialysis	Serum phosphate, >6.0 mg/dL	18 ms	LC (375–1500) CC (1000–3000)	50, 44/16 55, 38/17	63 ± 13 65 ± 14	NR NR
Gao, Y	2018	China	RCT	CKD 3–4, ND	Serum phosphate, >5.8 mg/dL	24 ms	LC (NR) CC (NR) OAC (NR)	16, NR 17, NR 17, NR	NR NR NR	NR NR NR
Ogata, H	2021	Japan	RCT	HD	Serum phosphate, >6.0 mg/dL	3.16 years	LC (750–2250) CC (3000)	1063, 633/430 1072, 638/434	69 (63, 75) 69 (63, 75)	4.8 (2.1, 9.4) 4.6 (2.0, 9.2)
Prajapati, VA	2014	India	Retrospective cohort	ESRD 5, HD	Serum phosphate, >6.5 mg/dL	12 weeks	LC (500) CC (500) CA (667) SH (400)	30, 16/14 30, 19/11 30, 12/18 30, 18/12	46.1 ± 12.5 46.03 ± 15.6 49.23 ± 17.3 49.73 ± 13.0	3.5 ± 1.1 ms 3.4 ± 1.0 ms 3.7 ± 1.0 ms 4.1 ± 0.9 ms
Sprague, SM (b)	2009	Multi-center^b^	RCT	CKD 5, HD	Serum phosphate, >6.0 mg/dL	4 weeks	LC (2250–3000)	181, 102/79	55.5 ± 13.1	3.22 ± 3.73
							SH (4800–6400)			
Finn, WF	2006	Multi-center^a^	RCT	ESRD, HD or CAPD	Serum phosphate, >5.9 mg/dL	2 years	LC (375–3000) Non-LC PBs	682, 390/292 677, 415/262	53.8 ± 14.6 54.9 ± 14.4	3.9 ± 3.4 3.8 ± 3.2
Wilson, R	2009	Multi-center^a^	RCT	ESRD, HD or CAPD	Serum phosphate, >5.9 mg/dL	2 years	LC (375–3000) Non-LC PBs	680, 389/291 674, 414/260	53.8 ± 14.5 54.9 ± 14.4	3.4 ± 3.4 3.3 ± 3.2
Kalil, RS	2012	USA	RCT	CKD 5, HD	Serum phosphate, >5.6 mg/dL	12 ms	LC (750–1500) Non-LC PBs	7, NR 6, NR	65 ± 9 68 ± 9	7.5 ± 5 3.7 ± 2
Komaba, H	2015	Japan	Retrospective cohort	HD	Serum phosphate, >6.0 mg/dL	3 years	LC (821 ± 358) Non-LC PBs	281, NR 263, NR	NR NR	NR NR
Hutchison, A	2018	USA	Retrospective cohort	ESRD, dialysis	NR	5 years	LC (NR) Non-LC PBs	2026, 1178/848 8094, 4706/3388	57.1 ± 14.6 56.6 ± 14.6	NR NR
Joy, MS	2003	USA	RCT	ESRD, HD	Serum phosphate, >5.9 mg/dL	4 weeks	LC (375–3000) Placebo	49, 32/17 44, 29/15	60.2 ± 13.3 60.5 ± 13.6	3.3 ± 3.2 3.0 ± 3.4
Finn, WF	2004	USA	RCT	ESRD, HD	Serum phosphate, >5.6 mg/dL	6 weeks	LC (2250) LC (1350) LC (675) LC (225) Placebo	26, 17/9 30, 17/13 29, 19/10 27, 14/13 32, 13/19	54 59.4 57.5 53.6 56.8	4.3 ± 3.7 3.1 ± 1.4 3.5 ± 3.0 3.5 ± 3.9 2.5 ± 1.18
Chiang, SS	2005	Taiwan	RCT	ESRD, HD	Serum phosphate, >5.6 mg/dL	4 weeks	LC (375–3000) Placebo	30, 16/14 31, 14/17	53.6 ± 11.2 51.7 ± 9.4	5.7 ± 3.4 5.3 ± 3.2
Shigematsu, T (b)	2008	Japan	RCT	CKD, HD	Serum phosphate, >5.6 mg/dL	6 weeks	LC (750) LC (1500) LC (2250) LC (3000) Placebo	30, 13/17 28, 21/7 31, 18/13 22, 17/5 31, 18/13	54.2 ± 9.6 58.6 ± 10.3 59.5 ± 8.6 60.0 ± 10.3 58.9 ± 9.9	9.8 ± 6.6 9.8 ± 5.3 8.8 ± 7.3 8.1 ± 4.6 9.3 ± 5.9
Sprague, SM (a)	2009	USA	RCT	CKD 3–4, HD	Serum phosphate, >4.6 mg/dL	8 weeks	LC (750–3000) Placebo	78, 40/38 41, 21/20	61.8 ± 12.9 63.0 ± 12.7	<4 ms <4 ms
Hutchison, AJ	2013	UK	RCT	CAPD	Serum phosphate, >5.58 mg/dL	4 weeks	LC (750–2250) Placebo	10, 6/4 11, 8/3	51.5 ± 17.5 54.4 ± 15.3	11.0 (6.0–87.0) ms 13.0(6.0–107.0) ms
Xu, J	2013	China	RCT	CKD 5D, HD or CAPD	Serum phosphate, >5.6 mg/dL	4 weeks	LC (1500–3000) Placebo	114, 60/54 113, 72/41	47.6 ± 13.0 48.4 ± 11.7	NR NR
Takahara, Y	2014	Japan	RCT	CKD 4–5, ND	Serum phosphate, >5.6 mg/dL	8 weeks	LC (750–2250) Placebo	86, 39/47 55, 28/27	61.3 ± 11.4 62.1 ± 12.8	NR NR
Wang, XH	2015	China	RCT	HD	Serum phosphate, >5.6 mg/dL	3 ms	LC (500) No PBs	28, 16/12 26, 15/11	68.9 ± 9.6 69.9 ± 10.9	2.8 ± 1.2 3.2 ± 1.3

M: male; F: female; ms, months; LC: lanthanum carbonate; ESRD: end-stage renal disease; HD: hemodialysis; CAPD: continuous ambulatory peritoneal dialysis; ND: non-dialysis; SH: sevelamer hydrochloride; CA: calcium acetate; CC: calcium carbonate; PBs: phosphate binders; DM: diabetic nephropathy; OAC: oral activated charcoal; RCT: randomized controlled trial; NR: not reported.

^a^USA, PuertoRico, Poland and South Africa.

^b^USA, Puerto Rico, Germany and the UK.

### 3.2. Quality assessment

**Figure 2.**
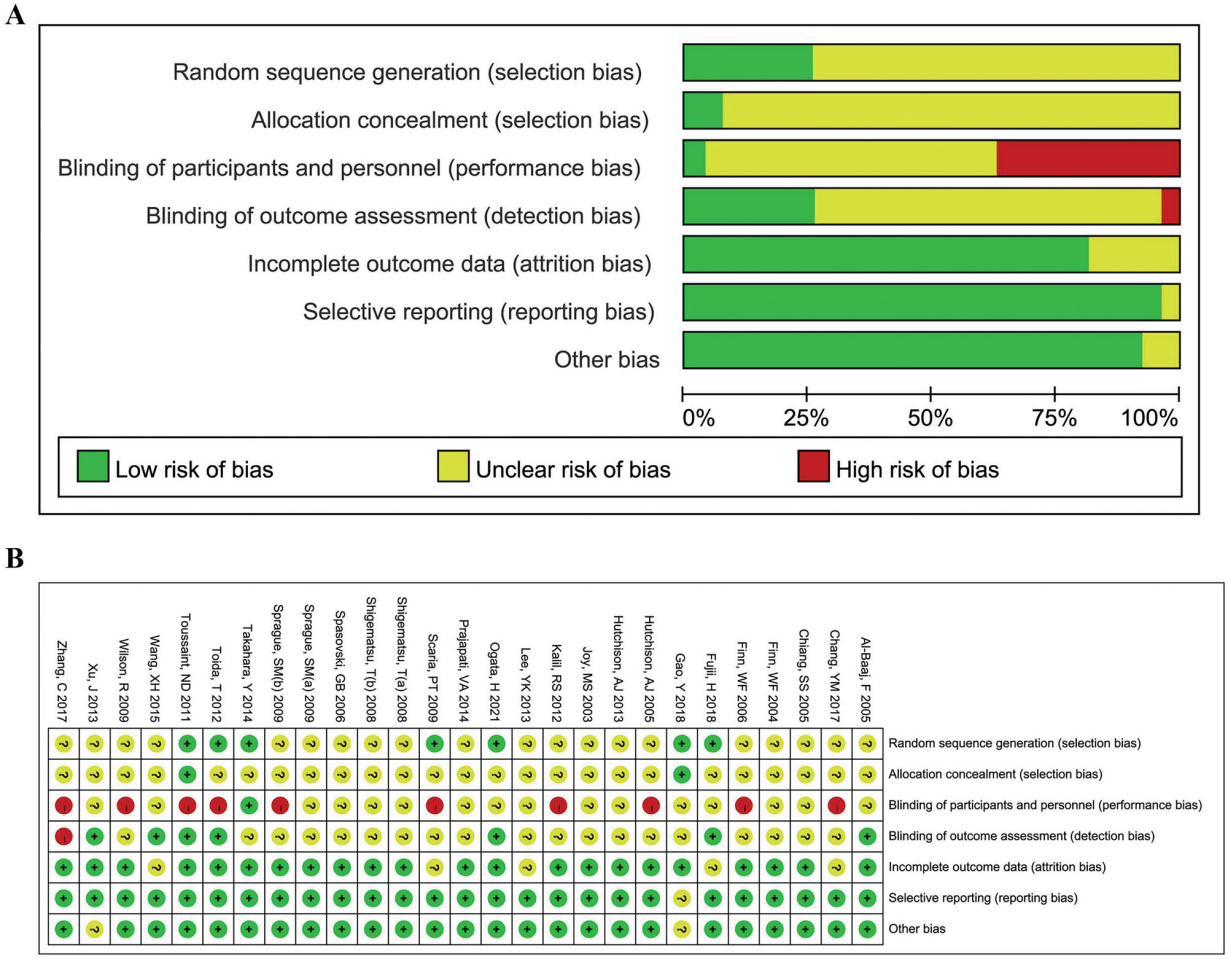
Quality assessments of the included randomized controlled trial (RCT) articles. (A) Risk of bias graph; (B) risk of bias summary for all RCT studies.

The results of the quality assessment are shown in Figure 2 and Supplementary Table 4. Due to most of the RCT did not elaborate the specific randomization, allocation concealment methods, and the method for the blind implementation, the degree of bias in the sequence generation, allocation concealment, blinding of participants and personnel, and blinding of outcome assessment were dominated by unclear outcomes. Besides, the bias in the incomplete outcome data, selective outcome reporting, and other biases was low risk. Overall, the RCT included in this study has a good methodological quality. The quality of the three retrospective cohort studies scored 6–7 and was also a moderate quality study.

### 3.3. Meta-analysis of pooled quantitative data

#### 3.3.1. Serum biochemical parameters

##### 3.3.1.1. Serum phosphorus control

**Figure 3.**
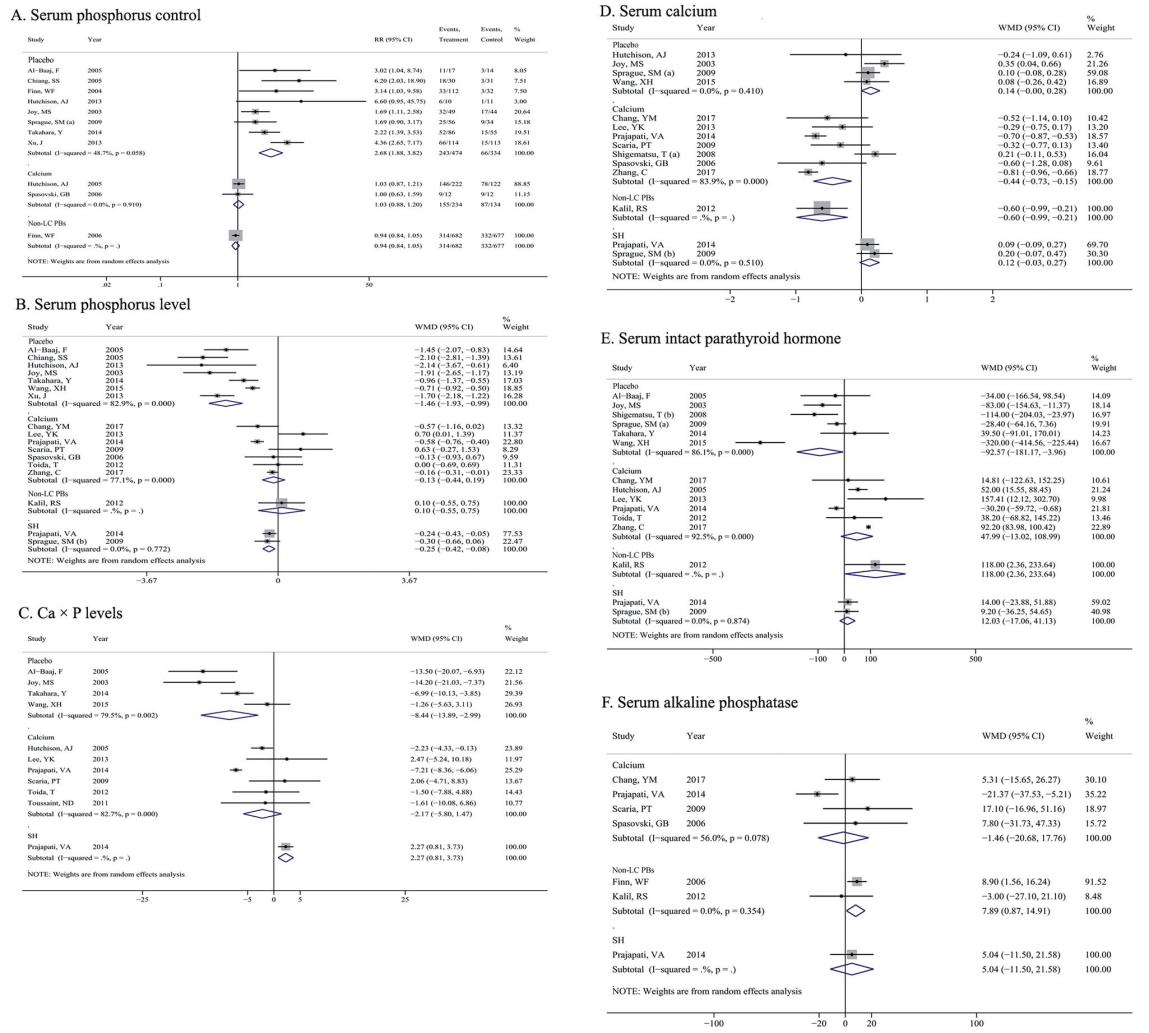
Pooled results for the serum biochemical parameters of LC treatment versus calcium salts, non-LC PBs, sevelamer, and placebo in patients with chronic kidney disease (CKD). (A) serum phosphorus control; (B) serum phosphorus; (C) Ca × P levels; (D) serum calcium; (E): serum intact parathyroid hormone; (F) serum alkaline phosphatase.

Serum phosphorus control refers to the achievement of standard serum phosphorus levels in the literature. As shown in Figure 3(A), there were 8, 2, and 1 studies that reported the serum phosphorus control comparison for LC vs. placebo, LC vs. Calcium, and LC vs. non-LC PBs. The combined results of LC vs. placebo were RR (95% CI) =2.68 (1.88, 3.82) and *p* < 0.001, without heterogeneity among studies (*I*^2^ = 48.7%, *p* = 0.058), indicating that LC had a better serum phosphorus control effect compared with placebo. However, no significant difference was observed among LC vs. calcium salts (RR = 1.03, 95% CI = 0.88 − 1.20; *p* = 0.750) or LC vs. non-LC PBs (RR = 0.94, 95% CI = 0.84 − 1.05; *p* = 0.269).

##### 3.3.1.2. Serum phosphorus level

As shown in Figure 3(B), there were 7, 7, 1, and 2 studies that reported the level of serum phosphate (mg/dL) comparison for LC vs. placebo, LC vs. calcium salts, LC vs. non-LC PBs, and LC vs. sevelamer. There was a significant decrease in serum phosphorus levels with LC in comparison with placebo (WMD= −1.46, 95%CI = −1.93, −0.99; *p* < 0.001), with considerable heterogeneity among studies (*I*^2^ = 82.9%, *p* < 0.001). Similarly, patients treated with LC had lower serum phosphorus levels compared with sevelamer (WMD= −0.25, 95%CI = −0.42, −0.08; *p* = 0.003), while no significant heterogeneity was found (*I*^2^ = 0.0%, *p* = 0.772).

##### 3.3.1.3. Ca × P product

As shown in Figure 3(C), there were 4, 6, and 1 studies that reported the Ca × P product comparison for LC vs. placebo, LC vs. calcium salts, and LC vs. sevelamer. There was a significantly lower Ca × P product in patients treated with LC in comparison with placebo (WMD= −8.44, 95%CI = −13.89, −2.99; *p* = 0.002), while a relatively higher Ca × P levels with LC in comparison to sevelamer (WMD= 2.27, 95%CI = 0.81, 3.73; *p* = 0.002) was detected, without significant heterogeneity among studies (*p* ≥ 0.05 or *I*^2^ ≤ 50%).

##### 3.3.1.4. Serum calcium

As shown in Figure 3(D), there were 4, 7, 1, and 2 studies reported in serum calcium (mg/dL) comparison for LC vs. placebo, LC vs. calcium salts, LC vs. non-LC PBs, and LC vs. sevelamer. Patients treated with LC had a relatively lower serum calcium level compared with calcium salts (WMD = −0.44, 95%CI = −0.73, −0.15, *p* = 0.003). However, no significant difference was identified among LC vs. placebo and LC vs. sevelamer groups.

##### 3.3.1.5. Serum intact parathyroid hormone (iPTH)

As shown in Figure 3(E), there were 6, 6, 1, and 2 studies reported in serum iPTH comparison for LC vs. placebo, LC vs. calcium salts, LC vs. non-LC PBs, and LC vs. sevelamer. Serum iPTH levels were significantly lower in patients treated with LC vs. placebo (WMD = −92.57, 95%CI = −181.17, −3.96, *p* = 0.041), while no significant difference was observed among LC vs. calcium salt (WMD = 47.99, 95%CI = −13.02, 108.99, *p* = 0.123) and LC vs. sevelamer (WMD = 12.03, 95%CI = −17.06, 41.13, *p* = 0.418) groups.

##### 3.3.1.6. Serum alkaline phosphatase (ALP)

As shown in Figure 3(F), there were 4, 2, and 1 studies reported in ALP comparison for LC vs. calcium salts, LC vs. non-LC PBs, and LC vs. sevelamer. A significantly higher levels of serum ALP was detected in patients treated with LC in comparison to non-LC PBs (WMD = 7.89, 95%CI = 0.87, 14.91, *p* = 0.028), without considerable heterogeneity between studies (*I*^2^ = 0.0%, *p* = 0.354).

### 3.4. Adverse events

#### 3.4.1. Treatment-related adverse events (TRAEs)

**Figure 4.**
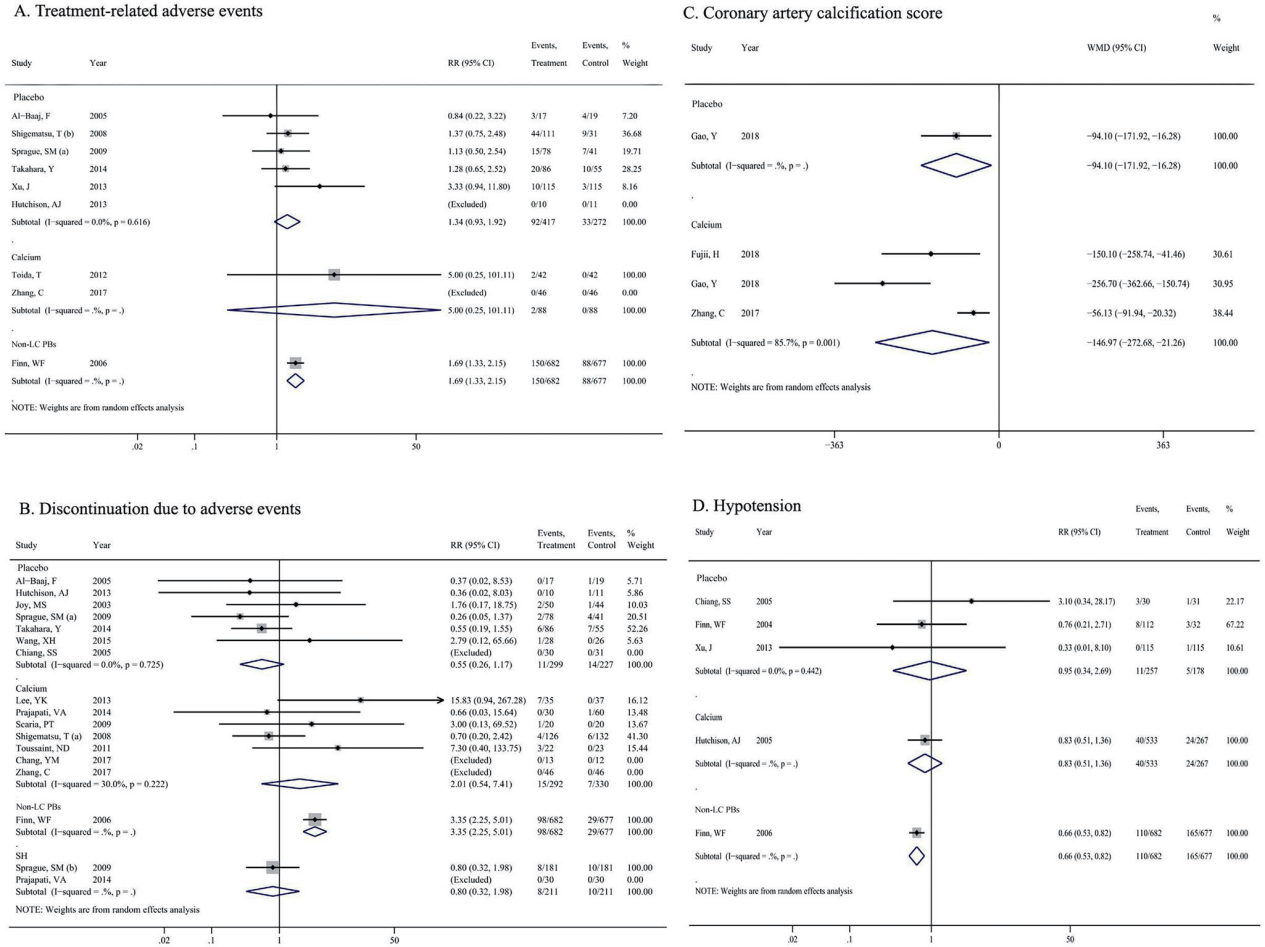
Pooled results for the major adverse events of LC treatment versus calcium salts, non-LC PBs, sevelamer, and placebo in CKD patients. (A) treatment-related adverse events; (B) discontinuation due to adverse events; (C) coronary artery calcification score; (D) hypotension.

There were no significant differences in the incidence of TRAEs *via* LC treatment induced in comparison to placebo (RR = 1.34, 95%CI = 0.93, 1.92; *p* = 0.112), and calcium salts (RR = 5.00, 95% CI = 0.25, 101.11; *p* = 0.294, Figure 4(A)). However, one study reported the effect of LC and non-LC PBs on the adverse events, and a significantly greater adverse events ratio was found in patients treated with LC vs. non-LC PBs (RR = 1.69, 95% CI = 1.33, 2.15; *p* < 0.001).

#### 3.4.2. Discontinuation due to adverse events (DtAEs)

Similarly, a significantly higher DtAEs with LC treatment compared with non-LC PBs was identified (RR = 3.35, 95% CI = 2.25, 5.01; *p* < 0.001, Figure 4(B)), while no remarkable difference existed among LC vs. placebo (*p* = 0.123), LC vs. calcium salts (*p* = 0.295), and LC vs. sevelamer groups (*p* = 0.630).

#### 3.4.3. Side effects of medications

Patients treated with LC had a lower score of coronary artery calcification in comparison to placebo (WMD = −94.10, 95%CI = –171.92, −16.28, *p* = 0.0188) or calcium (WMD = −146.97, 95%CI = –272.68, −21.26, *p* = 0.022, Figure 4(C)), a reduction ratio of hypotension (RR = 0.66, 95% CI = 0.53, 0.82; *p* < 0.001, Figure 4(D)) and abdominal pain (RR = 0.73, 95% CI = 0.59, 0.91; *p* = 0.004, Supplementary Figure 1A) compared with non-LC PBs, a decreased risk of diarrhea in comparison to placebo (RR = 0.32, 95% CI = 0.17, 0.60; *p* = 0.001) or non-LC PBs (RR = 0.75, 95% CI = 0.63, 0.90; *p* = 0.001, Supplementary Figure 1B), a reduced risk of dyspepsia (RR = 0.21, 95% CI = 0.07, 0.59; *p* = 0.003, Supplementary Figure 1C) and pruritus (RR = 0.15, 95% CI = 0.06, 0.37; *p* < 0.001, Supplementary Figure 1D) in comparison to placebo. No significant differences were observed in the risk of nausea (Supplementary Figure 1E), and vomiting (Supplementary Figure 1F).

### 3.5. Patient-level outcomes

#### 3.5.1. Mortality

**Figure 5.**
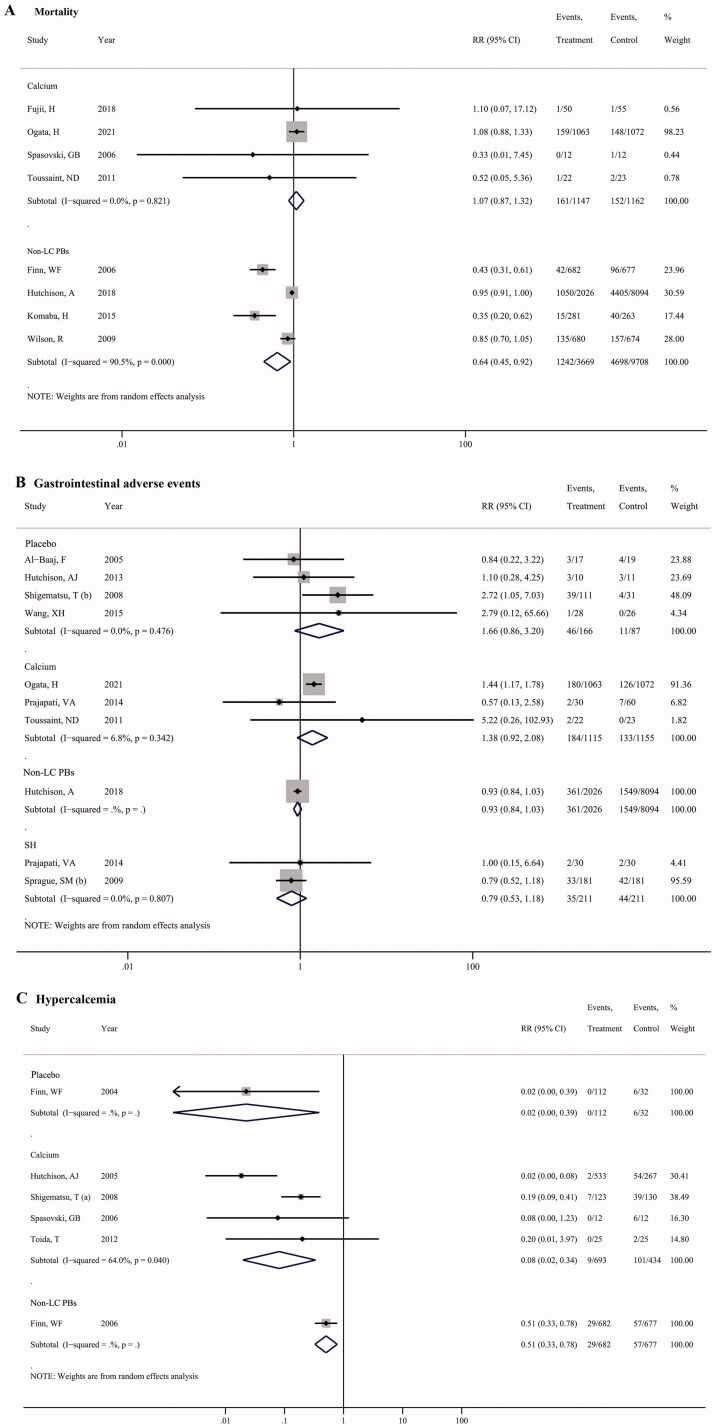
Pooled results for the patient-level outcomes of LC treatment versus calcium salts, non-LC PBs, sevelamer, and placebo in CKD patients. (A) mortality; (B) gastrointestinal adverse events; (C) hypercalcemia.

Meta-analysis of four studies showed a significantly lower risk in mortality with LC treatment in comparison to non-LC PBs (RR = 0.64, 95% CI = 0.45, 0.92; *p* = 0.016, Figure 5(A)). However, no significant difference in mortality risk was identified between LC and calcium salt groups (Figure 5(A)).

#### 3.5.2. Gastrointestinal adverse events

There was no significant difference in gastrointestinal adverse events with LC treatment in comparison to placebo, calcium salts, non-LC PBs, and sevelamer (Figure 5(B)), respectively.

#### 3.5.3. Hypercalcemia

There was a significant reduction risk in hypercalcemia with LC treatment in comparison with calcium salts based on meta-analysis of four studies (RR = 0.08, 95% CI = 0.02, 0.34; *p* = 0.001, Figure 5(C)). Similarly, only one study reported the hypercalcemia risk in patients treated with LC vs. placebo (RR = 0.02, 95% CI = 0.00, 0.39; *p* = 0.009), and LC vs. non-LC PBs (RR = 0.51, 95% CI = 0.33, 0.78; *p* = 0.002), and a decreased risk also detected in LC group.

### 3.6. Subgroup analysis

Subgroup analysis was further performed to explore the potential sources of heterogeneity. Factors for the subgroup analysis included age, area, and sample size. When patients were treated with LC compared with placebo, results showed that age, area, and sample size were significant effects on serum phosphate, and Ca × P outcomes (*p* < 0.05). In addition, when patients treated with LC were compared with calcium salts, results showed that age and area were significant effects on serum calcium, as well as age and sample size on hypercalcemia outcomes (*p* < 0.05; Table 2).

**Table 2.** Outcomes of subgroup analyze.

LC vs. others					
Outcomes	No. of studies	RR (95%CI) or WMD (95%CI)	*P*_A_	Heterogeneity test	
				*p*	*I*^2^ (%)
Phosphorus control					
Age					
≥60 years	3	1.87 (1.41, 2.47)	<0.001	0.658	0.0
<60 years	5	4.24 (2.89, 6.22)	<0.001	0.856	0.0
Area					
Asian	2	3.51 (1.96, 6.28)	<0.001	0.073	61.9
Western	6	1.94 (1.41, 2.66)	<0.001	0.476	0.0
Sample size					
≥100	3	3.09 (1.90, 5.04)	<0.001	0.149	47.5
<100	5	2.39 (1.47, 3.91)	<0.001	0.143	41.7
Serum phosphate					
Age					
≥60 years	3	–1.08 (–1.61, −0.55)	<0.001	0.007	79.7
<60 years	4	–1.74 (–2.06, −1.41)	<0.001	0.545	0.0
Area					
Asian	4	–1.31 (–1.90, −0.72)	<0.001	<0.001	87.9
Western	3	–1.68 (–2.14, −1.23)	<0.001	0.535	0.0
Sample size					
≥100	2	–1.32 (–2.04, −0.59)	<0.001	0.021	81.1
<100	5	–1.57 (–2.28, −0.86)	<0.001	<0.001	85.0
C × P					
Age					
≥60 years	3	–6.98 (–13.01, −0.96)	0.023	0.005	80.8
<60 years	1	–13.50 (–20.07, −6.93)	<0.001	––	––
Area					
Asian	2	–4.34 (–9.94, 1.26)	0.129	0.037	77.0
Western	2	–13.84 (–18.57, −9.10)	<0.001	0.885	0.0
Sample size					
≥100	1	–1.32 (–2.04, −0.59)	<0.001	––	––
<100	3	–9.36 (–18.53, −0.19)	0.045	0.001	86.3
Calcium					
Age					
≥60 years	3	0.15 (0.00, 0.30)	0.043	0.352	4.2
<60 years	1	–0.24 (–1.09, 0.61)	0.579	––	––
Area					
Asian	1	0.08 (–0.26, 0.42)	0.648	––	––
Western	3	0.16 (–0.05, 0.38)	0.132	0.254	27.0
Sample size					
≥100	1	0.10 (–0.08, 0.28)	0.285	––	––
<100	3	0.19 (–0.07, 0.44)	0.152	0.297	17.6
iPTH					
Age					
≥60 years	4	–99.20 (–225.03, 26.64)	0.122	<0.001	91.3
<60 years	2	–88.74 (–163.22, −14.27)	0.020	0.328	0.0
Area					
Asian	3	–135.44 (–328.85, 57.97)	0.170	<0.001	90.5
Western	3	–39.00 (–70.10, −7.90)	0.014	0.408	0.0
Sample size					
≥100	3	–40.82 (–108.55, 26.90)	0.237	0.114	0.539
<100	3	–148.12 (–319.06, 22.82)	0.089	<0.001	89.3
ALP					
Area					
Asian	3	–2.63 (–25.75, 20.49)	0.824	<0.001	90.5
Western	1	7.8 (–31.73, 47.33)	0.699	0.408	0.0
TRAEs					
Age					
≥60 years	2	1.21 (0.72, 2.05)	0.466	0.814	0.0
<60 years	4	1.49 (0.83, 2.68)	0.182	0.311	14.5
Area					
Asian	3	1.47 (0.96, 2.24)	0.073	0.400	0.0
Western	3	1.04 (0.52, 2.09)	0.910	0.713	0.0
Sample size					
≥100	4	1.39 (0.96, 2.02)	0.085	0.540	0.0
<100	2	0.84 (0.22, 3.22)	0.797	––	––
DtAEs					
Age					
≥60 years	4	0.59 (0.26, 1.30)	0.188	0.442	0.0
<60 years	3	0.37 (0.04, 3.32)	0.372	0.993	0.0
Area					
Asian	3	0.64 (0.24, 1.72)	0.378	0.337	0.0
Western	4	0.45 (0.14, 1.44)	0.179	0.634	0.0
Sample size					
≥100	2	0.45 (0.19, 1.07)	0.071	0.461	0.0
<100	5	0.99 (0.24, 4.18)	0.994	0.700	0.0
Nausea					
Age					
≥60 years	3	1.64 (0.54, 4.99)	0.382	0.230	32.0
<60 years	4	2.11 (0.34, 13.09)	0.423	0.050	61.7
Area					
Asian	3	9.74 (2.36, 40.13)	0.002	0.882	0.0
Western	4	0.78 (0.41, 1.48)	0.449	0.853	0.0
Sample size					
≥100	5	2.32 (0.69, 7.79)	0.173	0.032	62.2
<100	2	0.97 (0.21, 4.42)	0.966	0.477	0.0
Vomiting					
Age					
≥60 years	3	3.06 (1.06, 8.84)	0.039	0.965	0.0
<60 years	5	2.12 (0.53, 8.50)	0.290	0.077	52.6
Area					
Asian	4	4.62 (1.61, 13.26)	0.004	0.633	0.0
Western	4	0.88 (0.41, 1.81)	0.696	0.411	0.0
Sample size					
≥100	5	2.78 (0.74, 10.41)	0.130	0.030	62.6
<100	3	1.91 (0.48, 7.58)	0.357	0.881	0.0
Diarrhea					
Age					
≥60 years	2	0.40 (0.14, 1.16)	0.092	0.589	0.0
<60 years	3	0.28 (0.13, 0.62)	0.002	0.818	0.0
Area					
Asian	2	0.37 (0.11, 1.18)	0.092	0.688	0.0
Western	3	0.30 (0.14, 0.64)	0.002	0.696	0.0
Sample size					
≥100	3	0.29 (0.14, 0.58)	0.000	0.817	0.0
<100	2	1.91 (0.48, 7.58)	0.403	0.792	0.0
Constipation					
Age					
≥60 years	1	2.98 (0.90, 9.91)	0.074	––	––
<60 years	3	0.86 (0.20, 3.76)	0.838	0.807	0.0
Area					
Asian	3	1.95 (0.72, 5.29)	0.188	0.383	0.0
Western	1	1.10 (0.08, 15.36)	0.944	––	––
Sample size					
≥100	3	1.95 (0.72, 5.29)	0.188	0.383	0.0
<100	1	1.10 (0.08, 15.36)	0.944	––	––
GIAE					
Age					
≥60 years	1	2.79 (0.12, 65.66)	0.524	––	––
<60 years	3	1.57 (0.74, 3.30)	0.237	0.303	16.2
Area					
Asian	2	2.73 (1.10, 6.77)	0.030	0.988	0.0
Western	2	0.96 (0.37, 2.49)	0.933	0.780	0.0
Sample size					
≥100	1	2.72 (1.05, 7.03)	0.039	––	––
<100	3	1.05 (0.42, 2.62)	0.918	0.786	0.0

LC vs. calcium					
Outcomes	No. of studies	RR (95%CI) or WMD (95%CI)	*P*_A_	Heterogeneity test	
				*p*	*I*^2^ (%)
Serum phosphate					
Age					
≥60 years	1	0.00 (–0.69, 0.69)	1	––	––
<60 years	6	–0.14 (–0.48, 0.21)	0.437	<0.001	80.4
Area					
Asian	6	–0.12 (–0.46, 0.22)	0.495	<0.001	80.8
Western	1	–0.13 (–0.93, 0.67)	0.750	––	––
Sample size					
≥100	0	––	––	––	––
<100	7	–0.13 (–0.44, 0.19)	0.435	<0.001	77.1
C × P					
Age					
≥60 years	1	–1.50 (–7.88, 4.88)	0.645	––	––
<60 years	5	–2.24 (–6.26, 1.77)	0.274	<0.001	85.4
Area					
Asian	4	–1.73 (–7.52, 4.06)	0.559	0.002	80.2
Western	2	–2.19 (–4.23, −0.16)	0.035	0.889	0.0
Sample size					
≥100	1	–2.23 (–4.33, −0.13)	0.037	––	––
<100	5	–1.80 (–6.78, 3.19)	0.480	0.002	75.7
Calcium					
Age					
≥60 years	0	––	––	––	––
<60 years	7	–0.44 (–0.73, −0.15)	0.003	<0.001	83.9
Area					
Asian	6	–0.42 (–0.74, −0.11)	0.009	<0.001	86.6
Western	1	–0.60 (–1.28, 0.08)	0.082	––	––
Sample size					
≥100	1	0.21 (–0.11, 0.53)	0.193	––	––
<100	6	–0.64 (–0.81, −0.48)	<0.001	0.147	38.9
iPTH					
Age					
≥60 years	1	38.20 (–68.82, 145.22)	0.484	––	––
<60 years	5	49.69 (–17.20, 116.58)	0.145	<0.001	93.9
Area					
Asian	5	48.46 (–31.99, 128.92)	0.238	<0.001	93.7
Western	1	52.00 (15.55, 88.45)	0.005	––	––
Sample size					
≥100	5	52.00 (15.55, 88.45)	0.005	––	––
<100	1	48.46 (–31.99, 128.92)	0.238	<0.001	93.7
DtAEs					
Area					
Asian	6	1.60 (0.38, 6.76)	0.521	0.222	31.8
Western	1	7.30 (0.40, 133.75)	0.180	––	––
Sample size					
≥100	1	0.70 (0.20, 2.42)	0.571	––	––
<100	6	4.32 (0.96, 19.39)	0.056	0.504	0.0
Mortality					
Age					
≥60 years	2	1.08 (0.88, 1.33)	0.447	0.991	0
<60 years	2	0.44 (0.27, 2.87)	0.394	0.820	0
Area					
Asian	2	1.08 (0.88, 1.33)	0.447	0.991	0
Western	2	0.44 (0.27, 2.87)	0.394	0.820	0
Sample size					
≥100	2	1.08 (0.88, 1.33)	0.447	0.991	0
<100	2	0.44 (0.27, 2.87)	0.394	0.820	0
Constipation					
Area					
Asian	2	0.77 (0.31, 1.91)	0.566	0.946	0.0
Western	2	0.92 (0.53, 1.60)	0.772	0.440	0.0
Sample size					
≥100	2	0.86 (0.52, 1.41)	0.547	0.790	0.0
<100	2	1.05 (0.26, 4.32)	0.943	0.448	0.0
Hypercalcemia					
Age					
≥60 years	1	0.20 (0.01, 3.97)	0.291	––	––
<60 years	3	0.07 (0.01, 0.38)	0.002	0.017	75.6
Area					
Asian	2	0.19 (0.09, 0.40)	0.000	0.937	0.0
Western	2	0.02 (0.01, 0.09)	0.000	0.370	0.0
Sample size					
≥100	2	0.06 (0.01, 0.62)	0.018	0.004	87.7
<100	2	0.12 (0.02, 0.91)	0.041	0.646	0.0

*P*_A_: *p*-value for test of the association.

### 3.7. Publication bias test

Due to the number limitation of literature included in the comparison of other outcomes, the publication bias of blood phosphorus control and total AEs for LC vs. placebo were assessed. Results showed that no publication bias was identified in phosphorus control (*p* = 0.219) and total adverse events (*p* = 0.879).

## 4. Discussion

It remains a question regarding whether LC is a safe and efficacious treatment to patients with CKD. The present meta-analysis included 29 studies to examine the effect of LC in comparison to calcium salts, non-LC PBs, sevelamer, and placebo. Our results showed that LC can better reduce serum phosphorus levels and significantly reduce the risk of adverse events and mortality compared with other drugs, suggesting that LC may be a safe and effective PB for the treatment of hyperphosphatemia in CKD.

Hyperphosphatemia, which is related to increased incidence of cardiovascular disease, is a common complication in patients with CKD [45]. Serum phosphorus control is an important part of CKD treatment [13]. A large observational study reveals that serum phosphate and calcium-phosphate product (Ca × P) are independent risk factors for mortality in dialysis patients [46]. LC is a calcium-free PB, which is widely used in the management of dialysis patients [36]. Our systematic review identified that LC was superior to placebo, control diet, or other oral activated charcoal for serum phosphorus controls and reduction of Ca*×* P levels. In addition, a recent meta-analysis of patients with CKD treated by calcium or non-calcium-based PBs showed that calcium increased mortality in comparison with non-calcium-based PBs, such as sevelamer and LC [47]. Reportedly, emerging data suggest that calcium-containing agents have become somewhat limited due to the possibility of increasing the risk of vascular calcification and adynamic bone disease [48]. Furthermore, the large amount of calcium salts required for phosphate binding may affect their effectiveness for the related symptomatic hypercalcemia [46]. In this meta-analysis, no significant difference in mortality was identified between LC and calcium salt groups, while there was a significant reduction risk in hypercalcemia and coronary artery calcification with LC treatment in comparison to calcium salt group. Additionally, a remarkably lower risk in mortality with LC treatment in comparison to non-LC PBs was identified, and various adverse effects, such as hypotension, abdominal pain, diarrhea, and cough were reported with non-LC PBs in clinical trials. A previous study suggests that combination therapy with sevelamer and non-LC PBs is effective in decrease serum phosphate and Ca × P product in hyperphosphatemia patients, while gastrointestinal intolerance and compliance are significant side effects with such an approach [46]. LC is poorly absorbed from the gastrointestinal tract and mainly eliminated by the liver. A 6-year follow-up study reported that LC was not associated with adverse reactions in the liver of patients with CKD [49,50]. Taken together, LC might be a safe and effective agent for hyperphosphatemia treatment in patients with CKD.

In this meta-analysis, we did not focus on the effect of comorbid drugs on the phosphorus lowering effect of phosphorus binders because most articles did not list the comorbid drugs with phosphorus binders. Recent studies have found that proton pump inhibitors (PPI) significantly attenuate the phosphorus reduction effect of LC, although it does not significantly affect the phosphorus reduction effect of ferric citrate hydrate (FCH) and sucroric oxyhydroxide (SFOH) [51]. It has been reported that PPI also affects the phosphorus reducing effect of calcium [52]. This suggests that we need to pay attention to the effect of comorbid drugs on phosphorus binders in future similar meta-analyses.

The heterogeneity test showed an obvious heterogeneity among studies for some variables. Thus, the random effect model was chosen to assess the pooled effect. The significant heterogeneity might be related to the various baseline characteristics of enrolled patients. For instance, patients involved in this study come from diverse regions of the world, and the age distribution ranged from 6 to 83 years old, mostly with middle-aged patients. Moreover, different stages of patients with CKD who underwent different dialysis modalities were included, such as early stage of CKD, end-stage renal disease, hemodialysis, peritoneal dialysis, or non-dialysis. In addition, the sample size might be another reason for the observed heterogeneity. For instance, 2026 patients treated with LC and 8094 patients treated with non-LC PBs were registered in the study by Randen *et al*, whereas only 17 patients treated with calcium and 17 patients treated with oral activated charcoal were registered in the study by Hutchison et al. [24].

## 5. Conclusion

In conclusion, compared with calcium salts, non-LC PBs, sevelamer, and placebo, LC showed higher ability in serum phosphorus controls, reduced risk in gastrointestinal adverse events, coronary artery calcification, and mortality for patients with CKD. LC seemed to be a safe and effective agent in CKD treatment. However, future meta-analyses with a larger number of eligible primary articles still need to be carried out for more reliable results.

## 6. Limitations and perspectives

There are some limitations in this study: (1) publication bias test for most variables comparison except phosphorus control and total adverse events were not performed due to the less included studies; (2) the follow-up period included in the study were inconsistently ranged from four weeks to 5 years, and there was still no literature available to systematically assess the short-, medium- and long-term efficacy and safety of LC for CKD; (3) For some variables comparison, the less included study and small sample size might affect the results of meta-analysis, such as the comparison of total adverse events incidence between LC and non-LC PBs, only one study reported the effect of LC and non-LC PBs on the adverse events, and a significantly greater adverse events ratio was found with LC treatment. Hence, the analysis based on more studies with high quality was needed to confirm the clinical effect of LC in CKD treatment. In addition, this meta-analysis had not been registered online in advance, but the study was carried out and the article was written strictly according to the PRISMA statement.

## Supplementary Material

Supplemental Material

Supplemental Material
