# Supplementary figures and images for: Safety and effectiveness of lanthanum carbonate for hyperphosphatemia in chronic kidney disease (CKD) patients: a meta-analysis
Source: Ren Fail. 2021 Oct 4;43(1):1378–93. doi: 10.1080/0886022X.2021.1986068 (PMC8491672; doi:10.1080/0886022X.2021.1986068)

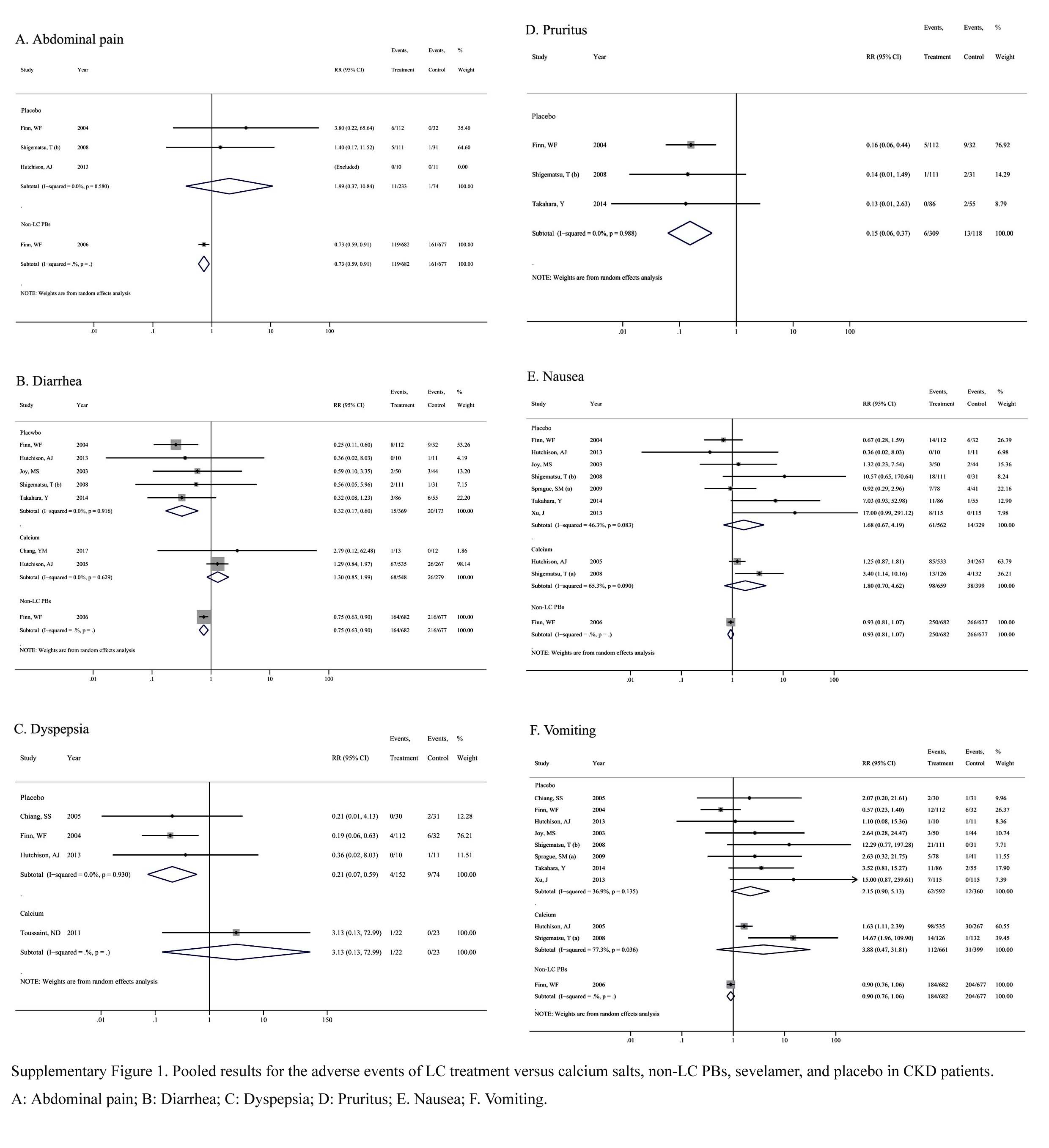

Supplement: Supplemental Material [file IRNF_A_1986068_SM0657.jpg]
