# Supplementary material for: Safety and effectiveness of lanthanum carbonate for hyperphosphatemia in chronic kidney disease (CKD) patients: a meta-analysis
Source: Ren Fail. 2021 Oct 4;43(1):1378–93. doi: 10.1080/0886022X.2021.1986068 (PMC8491672; doi:10.1080/0886022X.2021.1986068)
Supplement: Supplemental Material [file IRNF_A_1986068_SM0654.pdf]

The retrieval time: 20210618

Supplementary table 1 Results of pubMed retrieval

Search	Query	Items found
#1	"kidney failure, chronic"[MeSH Terms] OR "chronic kidney failure"[All Fields] OR "chronic renal failure"[All Fields]	105208
#2	"CKD"[All Fields] OR ("renal insufficiency, chronic"[MeSH Terms] OR "chronic renal insufficiency"[All Fields] OR "chronic kidney disease"[All Fields])	153432
#3	("chronic"[All Fields] OR "chronical"[All Fields] OR "chronically"[All Fields] OR "chronicities"[All Fields] OR "chronicity"[All Fields] OR "chronicization"[All Fields] OR "chronics"[All Fields]) AND ("kidney diseases"[MeSH Terms] OR "kidney diseases"[All Fields] OR "nephropathies"[All Fields] OR "nephropathy"[All Fields])	168694
#4	"haemodialysis"[All Fields] OR "renal dialysis"[MeSH Terms] OR "renal dialysis"[All Fields] OR "hemodialysis"[All Fields] OR ("peritoneal dialysis"[MeSH Terms] OR "peritoneal dialysis"[All Fields])	143836
#5	"end stage renal disease"[All Fields] OR "esrd"[All Fields]	38885
#6	#1 OR #2 OR #3 OR #4 OR #5	283313
#7	"lanthanum carbonate"[Supplementary Concept] OR "lanthanum carbonate"[All Fields] OR "fosrenol"[All Fields] OR ("dilanthanum"[All Fields] AND "tricarbonate"[All Fields])	546
#8	#6 AND #7	444

Supplementary table 2 Results of Embase retrieval

Search	Query	Items found
#1	(ckd OR 'chronic renal failure'/exp OR 'chronic renal failure' OR 'chronic kidney disease'/exp OR 'chronic kidney disease')	166494
#2	chronic AND ('nephropathy'/exp OR nephropathy)	226309
#3	('hemodialysis'/exp OR hemodialysis OR 'peritoneal dialysis'/exp OR 'peritoneal dialysis')	166400
#4	('end stage renal disease'/exp OR 'end stage renal disease' OR 'esrd'/exp OR esrd)	70298
#5	#1 OR #2 OR #3 OR #4	392754
#6	('lanthanum carbonate'/exp OR 'lanthanum carbonate' OR 'fosrenol'/exp OR fosrenol OR 'dilanthanum tricarbonate')	1149
#7	#5 AND #6	965

Supplementary table 3 Results of The Cochrane libraryretrieval

Search	Query	Items found
#1	MeSH descriptor: [Kidney Failure, Chronic] explode all trees	4767
#2	MeSH descriptor: [Renal Insufficiency, Chronic] explode all trees	6930
#3	MeSH descriptor: [Renal Dialysis] explode all trees	5219
#4	(CKD OR (chronic renal failure) OR (chronic kidney disease) OR (chronic nephropathy) OR hemodialysis OR (Peritoneal dialysis) OR (end stage renal disease) OR ESRD):ti,ab,kw(Word variations have been searched)	29730
#5	#1 OR #2 OR #3 OR #4	30276
#6	((Lanthanum carbonate) OR fosrenol OR (dilanthanum tricarbonate)):ti,ab,kw(Word variations have been searched)	264
#7	#5 AND #6	217
#8	#15 in Trials	215

Supplementarytable 4 Quality assessment of the cohort studies with Newcastle-Ottawa quality assessment scale

Study	Representati-veness of the exposed cohort	Selection of the unexposed cohort	Ascertainment of exposure	Outcome of interest not present at start of study	Control for important factor or additional factor	Outcome assessment	Follow-up long enough for outcomes to occur	Adequacy of follow-up of cohorts	Total quality scores
Prajapati, VA (2014)	☆	☆	☆	--	☆	☆	☆	☆	7
Komaba, H (2015)	☆	--	☆	--	☆	☆	☆	☆	6
Hutchison, A (2018)	☆	☆	☆	--	☆	☆	☆	☆	7
